# Carron Oil in Anal Fissure

**Published:** 1878-06

**Authors:** 


Carron Oil in Anal Fissure—This painful affection, which has heretofore resisted almost all forms of treatment by local application, has been successfully managed by Carrere, who states in *Annales de la Med. de Grand* that he applies the mixture of lime and water and linseed oil, so commonly used in burns. This is done several times daily, and in all cases he has obtained a cure of it at farthest, eight days.—*Allg. Med. Cent.-Zeit.*

